# Quality Characteristics of Substitute Meat Patties Developed Using *Aruncus dioicus* var. *kamtschaticus* Hara

**DOI:** 10.3390/foods11091341

**Published:** 2022-05-05

**Authors:** Kyung-Ok Shin, Hyo-Jeong Hwang, Kyoung-Sik Han, Yoo-Jin Lee

**Affiliations:** 1 Department of Food and Nutrition, Sahmyook University, 815 Hwarang-ro, Nowon-gu, Seoul 01795, Korea; hjhwang@syu.ac.kr (H.-J.H.); kshan@syu.ac.kr (K.-S.H.); 2 Department of Food Science and Biotechnology, Sahmyook University, 815 Hwarang-ro, Nowon-gu, Seoul 01795, Korea; mhjsea2000@naver.com

**Keywords:** *Aruncus dioicus* var. *kamtschaticus* Hara, patty, amino acid composition, chromaticity analysis, texture analysis

## Abstract

We developed a vegetable alternative to meat patties using *Aruncus dioicus* var. *kamtschaticus* Hara (*A. dioicus)* and used it to generate basic data for the alternative meat market by comparing nutritional and microbiological components with commercially available vegetable and meat patties. Nutrient analysis, microbiological analysis, chromaticity, and texture analysis were performed on substitute meat patties (SMPs) with *A. dioicus* and commercially available vegetable and animal patties. Among sugars, the contents of fructose and maltose were respectively high in commercial meat patties (CMPs) and SMPs. SMPs were low in saturated and trans-fat, and high in *ω*-3 fatty acids. The contents (in descending order) of leucine > phenylalanine > threonine > isoleucine were high in SMPs and commercial vegetable patties (CVPs). Qualitative and quantitative findings of *Escherichia coli (E. coli)* and *Staphylococcus aureus* were all negative. Our SMPs had high lightness (L*), low redness (a*), and low yellowness (b*). The hardness, chewiness, and resilience of our SMPs were lower than those of other vegetable and animal patties. Considering our results, the method of manufacturing SMPs developed in the present study allows meat to be flavored without significant nutritional differences compared with commercially available CMPs. Our findings provide a base for studies on future meat alternatives.

## 1. Introduction

Recent increased interest in family lifestyles has led to healthier eating habits, better health status, and expanded food choices. In the global context of COVID-19, contact rates between people have decreased, rates of staying home have increased, the amount of consumption has decreased, and the amount of physical activity has decreased. This has led to an increased prevalence of obesity, which is associated with an increasing incidence of colon cancer, breast cancer, cadiovascular disease, and high blood pressure [1,2,3]. According to a 1991 National Health and Nutrition Survey of Korean adults aged 20–29 years, 17.1% of obese people had a body mass index (BMI) of ≥25 kg/m^2^ [4]. By 2005, the prevalence of obesity among men aged ≥19 years had rapidly increased to 36.3% [5]. Interest in health has also affected food culture, and preferences for food choices, such as low-fat, low-salt, low-carbohydrate, and reducing excessive meat intake are increasing. In addition, studies of replacing animal fat in high-fat meat products are ongoing as trends in shares of the global substitute meat market are increasing.

Currently, plant-based meat substitutes, edible insects, and cultured meat are attracting attention as the most representative meat substitutes. Among them, plant-based alternatives to meat, representing the largest proportion, is a meat-like food manufactured using proteins extracted from plants. The main ingredients include wheat, pea protein, soybeans, peanuts, cottonseed, and rice [6,7]. The protein contents in soy flour, soy protein concentrate, and soy protein isolate are <50%, 70%, and 90%, respectively [8,9]. Soybean protein is a plant-based alternative that is mainly manufactured using textured vegetable protein (TVP) to give a texture similar to meat; it is consumed in Korea under the name “bean meat” [10]. According to Euromonitor, the market for alternative meat in the USA is increasing annually due to increases in sales of global companies such as “Impossible Food” and “Beyond Meat”. Thus, the estimated market for such products was $1.8 billion in 2015 and over $3 billion in 2020 [9]. To overcome future food shortages on this planet, we are constantly striving to replace the nutrients of animals with plants and to create the flavor of meat through plants. As an alternative, the importance of developing a health-centered alternative meat has been emphasized, and the market for alternative meat using various ingredients is increasing. The development of foods such as meat substitutes using alternative plant-based proteins and the energy sources necessary for humans by replacing meat intake through existing livestock breeding are urgently needed [9]. Therefore, a switch to a plant-based diet is needed in Korea. However, the texture, taste, smell, and method of cooking patties require further development before plant-based alternative meats could satisfy consumer tastes [9].

*Aruncus dioicus* var. *kamtschaticus* Hara is a perennial plant of the Rosaceae family that grows wild in alpine forests and propagates by seeds. The height is about 10–30 cm, the leaves are alternate, and the flowers are yellow-green [11]. It is cultivated for food in Ulleungdo as “samnamul” and for ornamental purposes. It is collected in the spring before the leaves open, and the tough part of the root is removed. It is consumed pickled, fried, or stir-fried [11,12,13,14]. *A. dioicus* is rich in saponins, which is helpful for treating chronic degenerative diseases, and young shoots are rich in carbohydrates and minerals. The whole plant is used medicinally because it can detoxify, prevent tonsillitis, exert antioxidant and antibacterial effects, and enhance stamina. In addition, *A. dioicus* is used when boiling beef soup [15]. Lipid accumulation is suppressed and insulin resistance is improved in C57BL/6 mice given a high-fat diet supplemented with *A. dioicus* [6,16]. *A. dioicus* is cytotoxic and has antioxidant activities against prostate cancer [17,18]. It has also caused weight loss in goats [19] and alleviated streptozotocin-induced diabetes in mice [20]. Aruncin B—a microtubule-damaging substance that activates apoptosis [21]—and the genome of *A. dioicus* chloroplasts have been analyzed [22]. *A. dioicus* inhibits the aging of UV-B-damaged skin [23]. However, little is known about the applications of *A. dioicus* in food nutrition.

Therefore, in this study, an alternative meat patty was developed using *A. dioicus*, a natural vegetable herb that has a meaty taste and chewy texture. This study was conducted to provide basic data for research on substitute meat by comparing the developed meat patty with existing commercially available products.

## 2. Materials and Methods

### 2.1. Ingredients of High-Protein Substitute Meat

Textured vegetable protein was hydrated for 2 h with water (*w*/*w*; distilled 6×) and then placed for 3 min in a W-110 dehydrator (Hanil Electric, Seoul, Korea). The premix comprising salt 8%, sugar 32%, onion powder 7%, garlic powder 4.1%, mushroom powder 19%, ginger powder 1%, coriander powder 1.5%, white pepper 1%, and pomegranate was evenly mixed with 10.3% powder and 16.1% sahmyook seasoning. The gravy (based on 100% oil) of meat patties was reproduced by mixing 0.7% methylcellulose HV, 43.9% coconut oil, 21.9% cocoa butter, and 0.7% sahmyook seasoning in hot water (32.9%) for 5 min using an HG-15A homogenizer (Daihan, Seoul, Korea). The homogenate was stored at −18 °C. The color of meat patties was replicated (based on 100% bitcocoa water) by homogenizing 2.5% cocoa powder and 7.5% beet powder in 90% water for 30 s. Dehydrated TVP (45%), water (12.5%), water-soluble smoked flavor (0.3%), lemon juice (0.3%), and bitcocoa water (5.8%) were ground in an SMX-9400MD blender (Shinil, Cheonan, Korea) for 1 min. The mixture and ground premix 5.1%; potato starch, 1.8%; mixed soy protein, 2.3%; methylcellulose HV, 1.2%; black pepper, 0.1%; xanthan gum, 0.2%; potassium chloride, 0.1%; smoke flavor powder, 0.5%; beef flavor powder, 0.3%; nutritional yeast flakes, 1.2%; oyster mushroom, 7%; and *A. dioicus* 3% were mixed and kneaded into a dough with canola oil (0.6%), barbecue flavor (0.7%), and fat-soluble smoked flavor (0.3%). The prepared oil (11.7%) was chopped and mixed, and then 80 ± 2 g of the dough was removed and molded into patties (content ratio determined in prior repeated experiments) (Table 1). These were frozen at −18 °C for 30 min, baked in a frying pan at 80–100 °C for 3 min per side, cooled to room temperature, then stored at 4 °C. Three types of raw patties were made (three of each), and all experiments were performed in triplicate.

### 2.2. Visual Assessment of Patties

**Figure 1:**
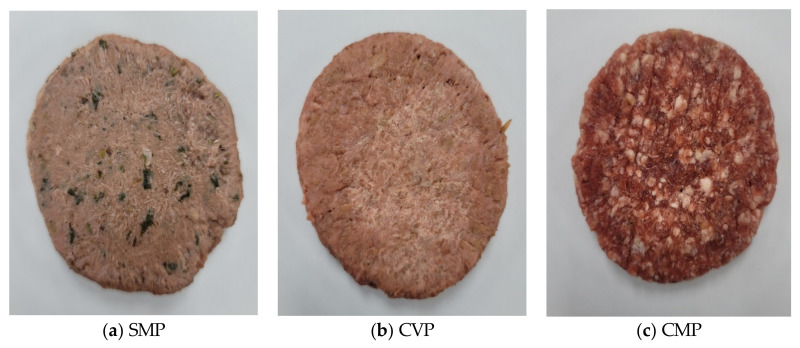
Overall side comparison of three patties. SMP—substitute meat patties developed using *A. dioicus*; CVP—commercial vegetable patty; CMP—commercial meat patty.

The entire surface of the raw patties was photographed using an Ev-nxflzza2qkr digital camera (Samsung, Suwon, Korea), and the characteristics (color, etc.) of the patties were visually assessed. Figure 1 shows the overall surfaces of the SMPs, CVPs and CMPs. The SMPs were lighter in color than the other two patties, and patties turned green after *A. dioicus* was added.

### 2.3. General Ingredients and pH Analyses

The moisture content of the *A. dioicus* was determined using an FS-620 atmospheric pressure dryer (Tokyo Seisakusho Co., Ltd., Osaka, Japan), and crude protein was analyzed by the micro-Kjeldahl method using a Kjeltec^TM^ 2300 automated Nitrogen analyzer (Foss Analytical AB., Hilleroed, Denmark). This was also measured according to the ash content by direct painting using a KL-160 (Toyo Seisakusho Co., Ltd., Osaka, Japan), and crude fat content was measured by the Soxhlet method using a SOX606 analyzer (Labtech, Seoul, Korea) [24,25]. The acidity/alkalinity of patties was measured using an HI 8014 pH meter (Hanna Instruments, Seoul, Korea) after suspending 10 g of dough in 50 mL of distilled water.

### 2.4. Free Sugar Analysis

Samples (1 g) were mixed with 40 mL of 80% ethanol at 100 rpm in a shaking incubator at 80 °C to extract free sugar as described in [26]. The mixture was filtered and centrifuged at 10,000× *g* for 20 min. The supernatant was passed through a 0.45 μm filter and analyzed using an LC-20AD HPLC (Shimadzu, Tokyo, Japan) with a carbohydrate column (4.6 × 250 nm, Waters Corp, Milford, MA, USA), with a mobile phase comprising acetonitrile in water (80:20, *v*/*v*) at a flow rate of 1.2 mL/min at 40 °C.

### 2.5. Analyses of Vitamin A, β-Carotene, Vitamin E, and Vitamin C

Vitamin A: A mixture of ethanol (30 mL) and 10% pyrogallol in ethanol (1 mL) was saponified with 3 mL of potassium hydroxide (9→10), in a reflux condenser placed in a boiling water bath for 30 min. The solution was cooled to room temperature in a flask, and then 30 mL of water was added and the mixture was passed through a brown separatory funnel. The flask was washed with 10 mL of water, followed by 30 mL of petroleum ether (special grade). The wash was added to a separatory funnel, shaken well, and then left to separate into layers. The water layer was extracted twice with 30 mL of petroleum ether in a brown separatory funnel. All petroleum ether extracts were combined and washed once with 10 mL of water, followed by 50 mL each (until the color did not change with phenolphthalein). Sodium sulfate was washed twice with 10 mL of petroleum ether, and the wash was added to the flask. All petroleum ether extracts were combined and evaporated to dryness under reduced pressure at 40–50 °C; the residue was dissolved in isopropanol (special grade) to 1.0 mL, serving as the test solution. The HPLC conditions were as follows: column; mobile phase, ethanol:water (95:5); flow rate, 0.5 mL/min; fluorescence detector with excitation and detection wavelengths of 340 and 460 nm, respectively.

β-carotene: Approximately 2 mg of β-carotene was dissolved in cyclohexane in 10 mL flasks, and then diluted with ethanol to prepare a standard solution. Portions (100–200 μg) of β-carotene were shaken with 10 mL of 3% pyrogallol in ethanol and 1 mL of 60% potassium hydroxide, and then saponified at 70 °C for 30 min. The saponified product was cooled with running water, and then shaken with 20 mL of 1% sodium chloride and 15 mL of hexane:ethyl acetate (9:1) for 10 min. After centrifugation at 2000 rpm for 5 min, the upper layer was concentrated, and 15 mL of hexane:ethyl acetate (9:1) was repeatedly added to the lower layer until the upper layer became colorless and the supernatants were combined. After concentration under reduced pressure, the solution was dissolved in 10 mL of ethanol to prepare test solutions. The LC-20AD HPLC conditions were as follows: Waters Atlantis dC18 column, C18 4.6 × 150 mm, 5 μm; mobile phase, 70% acetonitrile:methanol 85:15 (*v*/*v*; A) and 30% dichloromethane (B); flow rate, 1.0 mL/min; injection volume, 20 μL; run duration, 15 min; detector, 450 nm.

Vitamin E: Samples (~0.5 g) were saponified with 5 mL of 1 N KOH (in ethanol) containing a small amount of BHT and heated in capped 22 mL brown vials at 100 °C for 30 min. The vials were cooled at room temperature for ~15 min in the dark. Next, 5 mL of saturated saline and 10 mL of petroleum ether (containing a small amount of BHT) were manually shaken for at least 1 min, and then left for 10 min to separate the layers. Supernatants (5 mL) were placed in 20 mL tubes, and petroleum ether was removed at 40 °C using a Turbo-Vap evaporator (Caliper Life Science, Hopkinton, MA, USA). This was dissolved in 2 mL of methanol:isopropanol 1:1 (*v*/*v*), filtered with a 0.2 μm syringe filter, and then analyzed using the LC-20AD HPLC. The analytical conditions were as follows: column, LaChromUltra C18 (2 × 50 mm, 2 μm particle size); fluorescence detector, excitation and emission at 298 and 320 nm, respectively; flow rate, 0.2 mL/min; column oven temperature, 40 °C; isocratic elution with mobile phase, acetonitrile:methanol 50:50 (*v*/*v*); sample injection volume, up to 20 μL.

Vitamin C: Samples (~2 g if vitamin C content was ≥50 mg/100 g, and 5–10 g if 0–10 mg/100 g) were suspended in 10% methane phosphoric acid for 10 min, and then homogenized in 5% methane phosphoric acid. Homogenates were washed with a small amount of 5% methane phosphate, brought to 100 mL in volumetric flasks, and then centrifuged at 3000 rpm for 10–15 min. The supernatant was diluted with 5% methane phosphate to prepare test solutions that were analyzed by HPLC under the following conditions: column, YMC-Pack Polyamine II; mobile phase, 0.05 M KH_2_PO_4_:acetonitrile (60:40); flow rate, 1.0 mL/min; ultraviolet (UV) detector at 254 nm.

(1) $$\mathrm{Vitamin}\;C\;(\mathrm{mg}/100\;g)=\frac{S*a*b*100}{Sample\;\left(g\right)}\;\times \;1000$$

where *S* is the ascorbic acid concentration (μg/mL), *a* is the total amount (mL), and *b* is the dilution factor of test solutions.

### 2.6. Fatty Acid Composition Analysis

Samples (~25 mg) without fat were mixed with 2 mL of 0.5 N methanolic sodium hydroxide in stoppered glass tubes, heated for ~10 min at 100 °C, and then cooled. Trifluoroborane methanol (14%, 2 mL) was added; then, the mixture was heated at 100 °C for 10 min, cooled to 30–40 °C, and then vigorously shaken with 2 mL of isooctane for 30 s at this temperature. This mixture was shaken with 2 mL of saturated sodium chloride, cooled to room temperature, and then the isooctane layer was separated from the aqueous layer to serve as the test solution. The LC-20AD HPLC conditions were as follows: column, HP-FFAP (30 m × 0.32 mm ID; film thickness, 0.52 μm); oven temperature, 100 °C for 2 min, followed by 4 °C/min increments to 230 °C for 20 min; injector temperature, 230 °C; detector temperature, 250 °C; flow rate, 1.5 mL/min; and the instrument was set to GC-FID (Agilent 7890A) [27].

### 2.7. Amino Acid Composition Analysis

Amino acid content analysis was performed by modifying the method of Grunau and Swiader [28]. A 10 g amount of the sample was extracted three times with 150 mL of 70% ethanol at 80 °C for 2 h. To remove the fat-soluble component, hexane was added to extract again, the hexane fraction was removed, and the aqueous layer was concentrated under reduced pressure at 45 °C. The sample was concentrated in Uriprep (Waterbury, CT, USA), aliquoted in a 50 mL volumetric flask, centrifuged, and filtered with a 0.2 μm syringe filter. After about 40-fold dilution with lithium diluent (pH 2.36), 10 μL of the sample was injected into the Agilent 1100 Series (Agilent Technologies Inc., Santa Clara, CA, USA). For amino acid analysis, a cation exchange column (3 × 250 mm, 8 μm, Pickering Laboratories Inc., Mountain View, CA, USA) and PINNACLE PCX reactor (Pickering Laboratories Inc.) were used at a flow rate of 0.3 mL/min. Column and reactor temperatures were analyzed while maintaining 40 °C and 130 °C, respectively.

### 2.8. Qualitative Analysis of General Bacteria, Coliforms, and E. coli and Numbers of Staphylococcus aureus

Phosphate buffer solution (PBS) was prepared by dissolving 34 g of KH_2_PO_4_ in 500 mL of distilled water, adjusting the pH to 7.2 with 175 mL of 1 N NaOH, and then bringing the volume up to 1 L with distilled water. This stock buffer was sterilized by heating at 121 °C for 15 min, which was then stored at 4 °C. Immediately before use, 1 mL of the stock buffer was diluted in 800 mL of sterile phosphate buffered dilution water (Butterfield buffer). Samples were cut into small pieces using sterile scissors and tongs, and then 25 g of randomly selected pieces were homogenized in 225 mL of sterile butterfield buffer and used as test solutions. Plate count agar (PCA; tryptone, 5.0 g; yeast extract, 2.5 g; dextrose, 1.0 g; agar, 15.0 g) was dissolved in 1 L of distilled water adjusted to pH 7.0 ± 0.2 and then sterilized at 121 °C for 15 min. Test solutions (1 mL) were aseptically mixed with 1 mL of 10-fold Butterfield buffer in at least two sterile Petri dishes, and then PCA (~15 mL) was aseptically dispensed into petri dishes at 43–45 °C. The dishes were rotated gently and tilted from side to side to solidify the medium, taking care to avoid attachment to the lid. The development of diffuse colonies was suppressed by adding 3–5 mL of PCA and agitating the plates gently as described above. The petri dishes were inverted and incubated at 35 ± 1 °C for 48 ± 2 h. Colonies were counted in plates without diffuse colonies among 15–300 colonies per plate, and then the average number of colonies was multiplied by a dilution factor to calculate the number of bacteria (CFU/g).

Coliforms: Samples were finely cut using sterile scissors and tongs, and then random pieces (25 g) were homogenized in 225 mL of Butterfield buffer. Next, 1.0 and 0.1 mL of test solutions (as described for preparation in Brilliant green lactose bile broth (peptone, 10.0 g; lactose, 10.0 g; OxGall, 20.0 g; brilliant green, 0.0133 g)) in 1 L of distilled water were adjusted to a pH of 7.2 ± 0.1. Samples (10 mL) were dispensed into test tubes with fermentation tubes and sterilized at 121 °C for 15 min. Test solutions were incubated with BGLB medium at 35–37 °C for 48 ± 3 h, until gas was generated. Endo agar (peptone, 10.0 g; lactose, 10.0 g; dipotassium phosphate, 3.5 g; sodium sulfite, 2.5 g; basic fuchsin, 0.5 g; and agar, 15.0 g) was dissolved in 1 L of distilled water, adjusted to pH 7.4 ± 0.2, and sterilized at 121 °C for 15 min, or Eosin methylene blue agar (peptone, 10.0 g; lactose, 5.0 g; sucrose, 5.0 g; dipotassium phosphate, 2.0 g; eosin Y, 0.4 g; methylene blue, 0.065 g; and agar, 13.5 g) was dissolved in 1 L of distilled water, adjusted to pH 6.8 ± 0.2, and sterilized at 121 °C for 15 min. One typical or two or more atypical colonies in Endo agar or Eosin methylene blue agar were dissolved in nutrient agar (peptone, 5.0 g; beef extract, 3.0 g; and agar, 15.0 g) in 1 L of distilled water, adjusted to pH 6.8 ± 0.2, sterilized at 121 °C for 15 min, and incubated at 35–37 °C for 24 ± 2 h. Coliforms were identified in colonies cultured on nutrient agar using the VITEK 2 test Compact automated ID/AST instrument (bioMérieux SA., Marcy-l’Étoile, France).

*Escherichia coli*: Samples were cut finely using sterile scissors and tongs, and then randomly selected pieces (25 g) were homogenized in 225 mL of sterile Butterfield buffer. Test solutions (1 mL) according to preparation in 1 mL of distilled water with EC Broth (peptone, 20.0 g; lactose, 5.0 g; bile salt mixture, 1.5 g; dipotassium phosphate, 4.0 g; monopotassium phosphate, 1.5 g; and sodium chloride, 5.0 g) in 1 L of distilled water were adjusted to pH 6.9 ± 0.2. Next, 10 mL portions were dispensed into test tubes containing fermentation tubes, sterilized at 12 °C for 15 min, and then incubated at 44 ± 1 °C for 24 ± 2 h. Fermented material was inoculated onto EMB medium from the fermentations tubes and cultured at 35–37 °C for 24 ± 2 h, and then typical colonies were inoculated on ordinary agar medium and cultured at 35–37 °C for 24 ± 2 h. Colonies cultured on normal agar medium that were positive for *E. coli* were identified using VITEK 2 Compact.

Samples were into small pieces using sterile scissors and tongs, and then randomly selected pieces (25 g) in 225 mL of 10% NaCl were added to TSB medium (tryptone, 17 g; soytone, 3 g; dextrose, 2.5 g; sodium chloride, 5 g; and dipotassium phosphate, 2.5 g) in 1 L of distilled water, adjusted to pH 7.3 ± 0.2, and sterilized at 121 °C for 15 min. Enrichment culture proceeded at 35–37 °C for 18–24 h on Baird–Parker agar (tryptone, 10 g; beef extract, 5 g; yeast extract, 1 g; sodium pyruvate, 10 g; glycine, 12 g; lithium chloride 6H_2_O, 5 g; and agar 20 g) in 950 mL of distilled water, which was sterilized at 121 °C for 15 min, and then the pH was adjusted to 7.2. Glossy black colonies surrounded by transparent bands were placed on plain agar medium (5.0 g of peptone, 3.0 g of beef extract, and 15.0 g of agar were dissolved in 1 L of distilled water, pH 6.8 ± 0.2, which was sterilized at 121 °C for 15 min) and incubated at 35–37 °C for 18–24 h. *Staphylococcus aureus* was detected in cultured colonies using VITEK 2 Compact.

*Staphylococcus aureus* bacteria were counted as follows: Samples were cut into small pieces using sterile scissors and tongs, and 25 g of randomly selected pieces were homogenized in 225 mL of butterfield buffer. This served as the test solution. Test solutions were diluted 10-fold on three Baird–Parker agar plates (0.3, 0.4, and 0.3 mL) so that the total inoculum was 1 mL. The medium was completely dried to ensure that the inoculum was wholly absorbed into the medium and left for 10 min, before incubation at 35–37 °C for 48 ± 3 h; then, glossy black colonies surrounded by a transparent band were counted. Five or more typical colonies were selected from the counted plate, inoculated onto ordinary agar medium, and cultured at 35–37 °C for 18–24 h. Colonies positive for *S. aureus* were determined by multiplying the number of confirmed bacteria by the dilution factor.

### 2.9. Instrumental Color and Texture Analysis

Chromaticity was analyzed using a CR-400 Konica colorimeter (Minolta, Osaka, Japan). The L*, a*, and b* values were measured three times and used to determine the average value. At this time, L* = 94.8, a* = 0.0, and b* = 2.9 of the white plate used [29].

Analysis of the physical properties of the developed substitute meat patty product was measured using a texture analyzer (TAXT plus/50 Stable Micro Systems, Godalming, UK) for patties cut to a size of 2 cm × 2 cm × 2 cm. Analysis conditions were as follows: pretest speed, 2.0 mm/s; test speed, 1.0 mm/s; and posttest speed, 2.0 mm/s; and measured using a cylindrical probe. Hardness, adhesiveness, springiness, cohesiveness, chewiness, and resilience were measured three times and the average value was used [30].

### 2.10. Statistical Analysis

All experimental data are shown as the mean values with standard deviation and were statistically analyzed using SPSS version 18.0 (SPSS Inc., Chicago, IL, USA). Mean values were compared using one-way analysis of variance, and the significance of differences between them was analyzed using Duncan multiple range tests (*p* < 0.05).

## 3. Results and Discussions

### 3.1. General Ingredients, pH, Free Sugar Content, Vitamin Content, and Fatty Acid Composition

Table 2 shows that the general components and pH of SMPs, CVPs, and CMPs were as follows: moisture content, 60.59–62.48%; protein, 12.66–17.35%; fat, 6.08–16.50%; and raw material, 1.55–2.51%. The moisture content of SMPs was 62.48 ± 0.54%, which was similar to that of CVPs and CMPs. The protein contents of the SMPs (12.66 ± 0.10%) and CVPs (13.30 ± 0.29%) were also similar, whilst CMPs contained the most protein (17.35 ± 0.28%; *p* < 0.05). Fat content was highest in the order of CMPs, CVPs, and SMPs (16.50 ± 0.71%, 12.43 ± 0.17%, 6.08 ± 0.15%; *p* < 0.05). The crude content of CVPs was significantly higher among the three patties (2.51 ± 0.12%; *p* < 0.05). The pH range was 5.59–6.12; that of CMPs was low, at 5.59 ± 0.10. The protein contents of substitute meat patties containing leghemoglobin are similar to those of animal patties, but have lower fat and carbohydrate contents [9]. Crude fat and protein contents decrease in pork patties as the amount of added okara increases [31]. The pH range was 5.59–6.12, and the pH of CMPs was 5.59 ± 0.10. The protein content of SMPs developed with leghemoglobin is similar to that of animal patties, but the fat and carbohydrate contents are lower [9]. The crude fat and protein contents of pork patties decrease as the amount of added okara increases [31]. The moisture content of patties significantly increases as the amount of added cheonggukjang (fermented beans) increases [32]. In addition, the pH of pork patties with added green tea powder significantly increases as the amount of sample increases; the water content is the highest in patties with 2% added saltwort powder [33]. However, the moisture content of pork patties with added hemp and yulhui powders did not significantly differ with or without these powders or the rate at which they were added [34,35]. Furthermore, water holding capacity (WHC) decreases as the concentration of Methylcellulose (MC) increases (1.5–4%) in control and plant-based meat analog (PBMA) patties [36]. Therefore, [9,33,35], the difference in moisture content among patties with additives associated with the composition of raw meat is due to the type of sub-ingredients added during patty production. The difference is also dependent on the addition ratio and shape.

Table 2 shows the sugar content of three types of patties. The SMPs and CMPs contained 0.4 ± 0.01 and 0.5 ± 0.01 g/100 g of fructose per 100 g of patty (*p* < 0.05). We found 0.8 ± 0.01 g of glucose/100 g for SMPs and 0.1 ± 0.01 g/100 g for CVPs and CMPs, as well as 1.7 ± 0.01 g of sucrose and 0.7 ± 0.01 g of maltose/100 g for SMPs and 0.6 ± 0.01 g of sucrose/100 g for CVPs (*p* < 0.05).

Table 2 shows the vitamin contents of the three types of patties. Vitamin A accounted for 0.1 ± 0.01 g/100 g for CMPs, and β-carotene accounted for 0.3 ± 0.01 g/100 g for SMPs and 0.1 ± 0.01 g/100 g for CVPs (*p* < 0.05). Vitamin C was undetectable in the three types of patties. We detected 2.3 ± 0.01, 4.8 ± 0.01, and 1.0 ± 0.01 g of vitamin E/100 g for SMPs, CVPs, and CMPs, respectively (*p* < 0.05).

Table 2 shows the fatty acid composition of the three types of patties. The saturated fat contents were 7.9 ± 0.01, 6.9 ± 0.01, and 5.4 ± 0.01 g/100 g for CMPs, SMPs, and CVPs, respectively (*p* < 0.05). The trans fat and cholesterol contents were 81.6 ± 0.01 g/100 g for CMPs, but were undetectable in SMPs and CVPs (*p* < 0.05). Levels of *ω*-3 fatty acids were 64.0 ± 0.01, 55.8 ± 0.01, and 37.5 ± 0.01 g/100 g for SMPs, CVPs, and CMPs. Levels of *ω*-6 fatty acids were significantly higher (4984.0 ± 0.01 g/100 g for CVPs) than those of any other fatty acids (*p* < 0.05). In this study, trans fat and cholesterol content were not observed in SMPs and CVPs, but were high in CMPs at 81.6 ± 0.01 g per 100 g. *ω*-3 fatty acid and *ω*-6 fatty acid showed the highest levels in SMPs at 64.0 ± 0.01 g and 635.9 ± 0.01 g per 100 g, respectively. As the amount of okara added increases, total saturated fatty acids and cholesterol decrease in pork patties, and unsaturated fatty acids increase [31]. Park et al. [37] reported that when safflower seeds are added to ground pork as a fat substitute, the saturated fatty acid and cholesterol contents significantly decrease, while the unsaturated fatty acid content increases. In addition, saturated fatty acids decrease and unsaturated fatty acids increase in hamburger patties with added purple kohlrabi. Pinero et al. [38] reported that cholesterol decreases by ~6.0% when 13.4% of oat-soluble fiber is added to beef patties. Our findings were similar to these. Therefore, plant materials should be added to patties to reduce the content of cholesterol, which is harmful to health.

### 3.2. Amino Acid Composition

Table 3 shows the amino acid composition of the three types of patties. Except for phenylalanine, the content of essential amino acids was the highest in CMPs, and methionine was not detected in CVPs. The leucine content was higher for SMPs and CVPs at 919.4 ± 0.01 and 1099.2 ± 0.01 mg/100 g, respectively, whilst the lysine content was higher in CMPs at 1473.6 ± 0.01 mg/100 g (*p* < 0.05). In particular, the contents of the essential amino acids, leucine > lysine > phenylalanine > threonine > isoleucine, were high in SMPs and CVPs, whereas those of lysine > threonine > valine > phenylalanine > isoleucine were high in CMPs. The non-essential glutamic acid contents were 2452.1 ± 0.01, 2427.8 ± 0.01, and 2780.7 ± 0.01 mg/100 g for SMPs, CVPs, and CMPs, respectively (*p* < 0.05). Among the non-essential amino acids, all three patties contained glutamic acid > aspartic acid > arginine, but urea, L-hydroxyproline, sarcosine, L-citrulline, DL-2-aminobutyric acid, DL-3-aminoisobutyric acid, DL-plus-allo-δ-hydroxylysine, L-1-methylhistidine, L-3-methylhistidine, and L-carnosine were all undetectable. However, the L-amino acids contents were as follows: L-alanine (41.9 ± 0.01 mg/100 g), L-lysine (34.8 ± 0.01 mg/100 g), and L-leucine (33.3 ± 0.01 mg/100 g) for vegetable SMPs; L-alanine (54.0 ± 0.01 mg/100 g), L-glutamic acid (51.7 ± 0.01 mg/100 g), L-cystine (27.0 ± 0.01 mg/100 g), L-carnosine (141.4 ± 0.01 mg/100 g) for CVPs; and L-alanine (30.6 ± 0.01 mg/100 g) for CMPs. The content of taurine was the highest at 22.0 ± 0.01 mg/100 g of patty (*p* < 0.05). In this study, we judged that there is no significant difference in the amino acid composition of SMPs compared to CVPs. Amino acids are used to predict palatability by heating them with nonvolatile compounds such as peptides, amines, nucleic acids, sugars, and organic acids [9,39,40]. Aspartic and glutamic acids particularly contribute to umami when wine is added to pork patties [41]. In the study of pork patties with added purple kohlrabi [38], the most prevalent amino acids were (in order) glutamic acid, aspartic acid, lysine, and leucine. The essential amino acids in a meat substitute with leghemoglobin from soybean root nodules were leucine, valine, and isoleucine, and the non-essential amino acids were glutamine, glycine, and alanine; the amino acid composition was similar to that of commercially available animal patties [9].

### 3.3. Qualitative Analysis of General Bacteria, Coliforms, and E. coli and Numbers of Staphylococcus aureus

Table 4 shows bacterial analyses of the three types of patties. The number of normal bacteria was 43,000,000 colony forming units (CFU)/g in SMPs, whilst the maxima in CVPs and CMPs were 30,000 and 300 CFU/g, respectively. The coliform group was positive in SMPs, but *E. coli* was undetectable in all three types of patties in the qualitative evaluation. In addition, qualitative and quantitative findings of *Staphylococcus aureus* were also negative in all three patties. The total numbers of bacteria and numbers of coliforms significantly increase in pork patties with added parsley powder depending on the storage period, but these values do not significantly differ among groups with additives [42]. The total number of microorganisms in pork patties with added purple kohlrabi significantly increases over time in storage [40], and Reagan et al. [43] found that meat is edible when the total number of microorganisms is ≤6 log CFU/g.

### 3.4. Instrumental Color and Texture

Table 5 shows the chromaticity findings for SMPs, CVPs, and CMPs. The SMPs were significantly brighter than CMPs and CVPs (*p* < 0.05). The L* values were 34.10 ± 1.45, 33.30 ± 0.50, and 27.90 ± 1.77, in SMPs, CVPs, and CMPs, respectively (*p* < 0.05). The a* of the three types of patties was significantly higher in CMPs (9.80 ± 0.17) than that in SMPs and CVPs (7.50 ± 0.10 and 8.90 ± 0.63, respectively; *p* < 0.05). For b*, SMPs, CVPs, and CMPs were irradiated at 8.70 ± 0.30, 10.30 ± 0.45, and 10.40 ± 1.32, respectively (*p* < 0.05). The b value was the lowest in SMPs. Substitute meat patties developed by adding leghemoglobin have low lightness and yellowness but high redness [9]. Lightness decreases whilst redness and yellowness increase in patties with added kimchi powder [44]. Adding increasing amounts of saltwort powder to pork patties decreases lightness and redness and increases yellowness [32]. The color of patties is affected by heat and the types and amounts of additives [9,33].

Table 5 shows the physical properties of the SMPs, CVPs, and CMPs. The hardness of SMPs (365.68 ± 84.84) was significantly lower than that of CMPs (1026.23 ± 123.97) and CVPs (501.81 ± 19.34) that are currently on sale (*p* < 0.05). Values for physical properties such as chewiness and resilience were significantly higher for CVPs and CMPs than those for SMPs (*p* < 0.05). However, springiness and cohesiveness did not significantly differ among the three patties. The hardness, chewiness, and resilience of SMPs were lower than those of CVPs and CMPs. In SMPs developed by adding leghemoglobin, physical properties such as hardness, resilience, cohesiveness, and chewiness, excluding resilience, did not significantly differ among CVPs and animal patties. In particular, adding dietary fiber affected the physical properties of SMPs by increasing water retention [9]. The hardness and chewiness of the patties were increased by adding seaweed [45], and Kassama et al. [46] reported that adding texturized soy protein powder (TPS) to beef patties made them harder and more cohesive than the addition of soy protein powder. Adding saltwort powder to pork patties decreases hardness, chewiness, and gumminess depending on the amount added [33]. However, hardness, chewiness, gumminess, and cohesiveness significantly increase according to the amount of yulhui powder added in a study in which yulhui powder was added to pork patties [35].

## 4. Conclusions

In this study, a substitute meat patty was developed using *A. dioicus*. Considering the results of this study, it is judged that there is no significant difference in amino acid composition in SMPs compared to CVPs. The results of the qualitative evaluation of *E. coli* and the qualitative and quantitative evaluation of *Staphylococcus aureus* were all negative. The chromaticity of the developed patties was high in L*, and low in a* and b*. The hardness, chewiness, and resilience of the developed substitute meat patties were observed to be lower than those of vegetable and animal patties. It is believed that the results of this study can provide good basic data for future research on meat alternatives.

## Figures and Tables

**Table 1:** Patty manufacturing recipe using high protein alternative meat material.

Ingredients (%)	Substitute Meat Patty
TVP	45
TSP	2.3
Starch	1.8
Premix	5.1
Methylcellulose HV	1.2
Black pepper	0.1
Xanthan gum	0.1
Potassium chloride	0.1
Smoke flavor powder	0.5
Canola oil	0.6
Beef flavor powder	0.3
Nutritional yeast flakes	1.2
Water	12.5
Smoke flavor	0.3
Lemon juice	0.3
Barbecue flavor	0.7
Smoked products flavor	0.3
Beet powder and cocoa powder	5.8
Oil (water + Methylcellulose HV + coconut oil + cocoa utter + sahmyook seasoning)	11.7
Oyster mushroom	7
*Aruncus dioicus* var. *kamtschaticus* Hara	3
Total	100

TSP, textured soybean protein; TVP, textured vegetable protein.

**Table 2:** General ingredients, sugar content, fat-soluble vitamin content, and fatty acid composition of three patties.

Composition	Three Types of Patties		
	SMP	CVP	CMP
General ingredients (%)			
Moisture	62.48 ± 0.54 ^a^	60.59 ± 1.00 ^ab^	62.40 ± 2.77 ^a^
Protein	12.66 ± 0.10 ^b^	13.30 ± 0.29 ^b^	17.35 ± 0.28 ^a^
Fat	6.08 ± 0.15 ^c^	12.43 ± 0.17 ^b^	16.50 ± 0.71 ^a^
Ash	1.76 ± 0.08 ^b^	2.51 ± 0.12 ^a^	1.55 ± 0.11 ^b^
pH	6.12 ± 0.06 ^a^	6.39 ± 0.07 ^a^	5.59 ± 0.10 ^ab^
Ingredients (g/100 g)			
Fructose	0.4 ± 0.01 ^b^	0.0 ± 0.00 ^c^	0.5 ± 0.01 ^a^
Glucose	0.8 ± 0.01 ^a^	0.1 ± 0.01 ^b^	0.1 ± 0.01 ^b^
Lactose	0.0 ± 0.00	0.0 ± 0.00	0.0 ± 0.00
Saccharose	1.7 ± 0.01 ^a^	0.6 ± 0.01 ^b^	0.0 ± 0.01 ^c^
Maltose	0.7 ± 0.01 ^a^	0.0 ± 0.01 ^b^	0.0 ± 0.01 ^b^
Vitamin A	-	-	0.1 ± 0.01
Vitamin C	0.0 ± 0.00	0.0 ± 0.00	0.0 ± 0.00
Vitamin E	2.3 ± 0.01 ^b^	4.8 ± 0.01 ^a^	1.0 ± 0.01 ^c^
β-carotene	0.3 ± 0.01 ^a^	0.1 ± 0.01 ^b^	-
Saturated fat	6.9 ± 0.01 ^b^	5.4 ± 0.01 ^c^	7.9 ± 0.01 ^a^
Trans fat	0.0 ± 0.00 ^b^	0.0 ± 0.00 ^b^	0.6 ± 0.01 ^a^
Cholesterol	0.0 ± 0.00 ^b^	0.0 ± 0.00 ^b^	81.6 ± 0.01 ^a^
*ω*-3 fatty acid	64.0 ± 0.01 ^a^	55.8 ± 0.01 ^b^	37.5 ± 0.01 ^c^
*ω*-6 fatty acid	635.9 ± 0.01 ^b^	4,984.0 ± 0.01 ^a^	225.3 ± 0.01 ^c^

Values are means ± SD *(n* = 3); ^a–c^ means in a row with different letters are significantly different at *p* < 0.05, as analyzed by Duncan multiple range tests. SMP—substitute meat patties developed using *A. dioicus*; CVP—commercial vegetable patty; CMP—commercial meat patty.

**Table 3:** Essential amino acid and non-essential amino acid composition of three patties.

Ingredients (mg/100 g)	Three Types of Patties		
	SMP	CVP	CMP
Essential amino acids			
Threonine	471.0 ± 0.01 ^c^	515.2 ± 0.01 ^b^	792.8 ± 0.01 ^a^
Valine	436.7 ± 0.01 ^c^	507.3 ± 0.01 ^b^	673.2 ± 0.01 ^a^
Methionine	84.1 ± 0.01 ^b^	-	278.8 ± 0.01 ^a^
Isoleucine	454.8 ± 0.01 ^c^	531.4 ± 0.01 ^b^	607.2 ± 0.01 ^a^
Leucine	919.4 ± 0.01 ^c^	1099.2 ± 0.01 ^b^	1244.1 ± 0.0 ^a^
Phenylalanine	617.1 ± 0.01 ^b^	732.7 ± 0.01 ^a^	615.3 ± 0.01
Lysine	710.4 ± 0.01 ^c^	949.7 ± 0.0 ^b^	1473.6 ± 0.01 ^a^
Histidine	281.1 ± 0.01 ^c^	313.6 ± 0.01 ^b^	537.2 ± 0.01 ^a^
Non-essential amino acids			
Aspartic acid	1260.1 ± 0.01 ^b^	1590.7 ± 0.01 ^a^	1570.1 ± 0.01 ^a^
Serine	693.8 ± 0.01 ^b^	731.7 ± 0.01 ^a^	734.2 ± 0.01 ^a^
Glutamic acid	2452.1 ± 0.01 ^b^	2427.8 ± 0.01 ^b^	2780.7 ± 0.01 ^a^
Proline	486.1 ± 0.01 ^b^	576.0 ± 0.01 ^a^	572.6 ± 0.01 ^a^
Glycine	485.1 ± 0.01 ^c^	545.7 ± 0.01 ^b^	1004.2 ± 0.01 ^a^
Alanine	545.9 ± 0.01 ^c^	643.0 ± 0.01 ^b^	1078.3 ± 0.01 ^a^
Tyrosine	352.4 ± 0.01 ^c^	521.1 ± 0.01 ^a^	479.2 ± 0.01 ^b^
Arginine	729.6 ± 0.01 ^b^	1009.9 ± 0.01 ^a^	1089.1 ± 0.01 ^a^
L-amino acids			
Taurine	2.2 ± 0.01 ^c^	4.1 ± 0.01 ^b^	22.0 ± 0.01 ^a^
L-serine	14.9 ± 0.01 ^a^	7.5 ± 0.01 ^b^	5.3 ± 0.01 ^c^
L-glutamic acid	30.1 ± 0.01 ^b^	51.7 ± 0.01 ^a^	3.0 ± 0.01 ^c^
L-alanine	41.9 ± 0.01 ^b^	54.0 ± 0.01 ^a^	30.6 ± 0.01 ^c^
L-valine	14.1 ± 0.01 ^a^	11.4 ± 0.01 ^b^	3.8 ± 0.01 ^c^
L-cystine	-	27.0 ± 0.01	-
L-isoleucine	13.8 ± 0.01 ^a^	8.0 ± 0.01 ^b^	2.9 ± 0.01 ^c^
L-leucine	33.3 ± 0.01 ^a^	14.7 ± 0.01 ^b^	6.1 ± 0.01 ^c^
L-tyrosine	10.9 ± 0.01 ^a^	4.9 ± 0.01 ^b^	3.1 ± 0.01 ^c^
L-phenylalanine	27.5 ± 0.01 ^a^	8.3 ± 0.01 ^b^	3.5 ± 0.01 ^c^
β-alanine	12.8 ± 0.01 ^a^	1.8 ± 0.01 ^b^	-
L-lysine	34.8 ± 0.01 ^a^	8.6 ± 0.01 ^b^	4.6 ± 0.01 ^c^

Values are means ± SD (*n* = 3); ^a–c^ means in a row with different letters are significantly different at *p* < 0.05, as analyzed by Duncan multiple range tests. SMP—substitute meat patties developed using *A. dioicus*; CVP—commercial vegetable patty; CMP—commercial meat patty.

**Table 4:** Bacterial analysis of three patties.

Ingredients (CFU/g)	Three Types of Patties		
	SMP	CVP	CMP
Number of general bacteria	43,000,000	30,000	300
Coliform group (Quality)	Positive	Negative	Negative
Escherichia coli qualitative test	Negative	Negative	Negative
Staphylococcus aureus (Quality)	Negative	Negative	Positive

SMP—substitute meat patties developed using *A. dioicus*; CVP—commercial vegetable patty; CMP—commercial meat patty.

**Table 5:** Instrumental color and texture analysis of three patties.

	Three Types of Patties		
	SMP	CVP	CMP
Instrumental color analysis			
L (lightness)	34.10 ± 1.45 ^a^	33.30 ± 0.50 ^ab^	27.90 ± 1.77 ^b^
a (redness)	7.50 ± 0.10 ^c^	8.90 ± 0.63 ^b^	9.80 ± 0.17 ^a^
b (yellowness)	8.70 ± 0.30 ^b^	10.30 ± 0.45 ^a^	10.40 ± 1.32 ^a^
Texture analysis			
Hardness (g)	365.68 ± 84.84 ^c^	501.81 ± 19.34 ^b^	1026.23 ± 123.97 ^a^
Adhesiveness (gs)	−122.56 ± 3.89	−8.87 ± 3.45	−23.38 ± 3.12
Springiness (mm)	1.00 ± 0.00	1.00 ± 00	1.00 ± 0.00
Cohesiveness (g/s)	2.43 ± 0.63	2.72 ± 0.25	2.34 ± 0.04
Chewiness (g)	923.01 ± 456.01 ^c^	1376.00 ± 183.50 ^b^	2398.80 ± 248.77 ^a^
Resilience (g)	0.04 ± 0.00 ^c^	0.05 ± 0.00 ^b^	0.09 ± 0.00 ^a^

Values are means ± SD (*n* = 3); ^a–c^ means in a row with different letters are significantly different at *p* < 0.05, as analyzed by Duncan multiple range tests. SMP—substitute meat patties developed using *A. dioicus*; CVP—commercial vegetable patty; CMP—commercial meat patty.

## Data Availability

Data is contained within the article.
